# AIM2 nuclear exit and inflammasome activation in chronic obstructive pulmonary disease and response to cigarette smoke

**DOI:** 10.1186/s12950-021-00286-4

**Published:** 2021-05-22

**Authors:** Hai B. Tran, Rhys Hamon, Hubertus Jersmann, Miranda P. Ween, Patrick Asare, Rainer Haberberger, Harshita Pant, Sandra J. Hodge

**Affiliations:** 1 Department of Thoracic Medicine, Royal Adelaide Hospital, Adelaide, South Australia; 2 School of Medicine, University of Adelaide, Adelaide, South Australia; 3 Centre for Cancer Biology, University of South Australia and SA Pathology, Adelaide, South Australia; 4 Department of Anatomy and Histology, Flinders University of South Australia, Adelaide, South Australia

**Keywords:** AIM2 inflammasome, AIM2 protein nuclear localization, COPD, Cigarette smoke

## Abstract

**Introduction:**

The role inflammasomes play in chronic obstructive pulmonary disease (COPD) is unclear. We hypothesised that the AIM2 inflammasome is activated in the airways of COPD patients, and in response to cigarette smoke.

**Methods:**

Lung tissue, bronchoscopy-derived alveolar macrophages and bronchial epithelial cells from COPD patients and healthy donors; lungs from cigarette smoke-exposed mice; and cigarette smoke extract-stimulated alveolar macrophages from healthy controls and HBEC30KT cell line were investigated. AIM2 inflammasome activation was assessed by multi-fluorescence quantitative confocal microscopy of speck foci positive for AIM2, inflammasome component ASC and cleaved IL-1β. Subcellular AIM2 localization was assessed by confocal microscopy, and immunoblot of fractionated cell lysates. Nuclear localization was supported by in-silico analysis of nuclear localization predicted scores of peptide sequences. Nuclear and cytoplasmic AIM2 was demonstrated by immunoblot in both cellular fractions from HBEC30KT cells.

**Results:**

Increased cytoplasmic AIM2 speck foci, colocalized with cleaved IL-1β, were demonstrated in COPD lungs (*n* = 9) vs. control (*n* = 5), showing significant positive correlations with GOLD stages. AIM2 nuclear-to-cytoplasmic redistribution was demonstrated in bronchiolar epithelium in cigarette-exposed mice and in HBEC30KT cells post 24 h stimulation with 5% cigarette smoke extract. Alveolar macrophages from 8 healthy non-smokers responded to cigarette smoke extract with an > 8-fold increase (*p* < 0.05) of cytoplasmic AIM2 and > 6-fold increase (*p* < 0.01) of colocalized cleaved IL-1β speck foci, which were also localized with ASC.

**Conclusion:**

The AIM2 inflammasome is activated in the airway of COPD patients, and in response to cigarette smoke exposure, associated with a nuclear to cytoplasmic shift in the distribution of AIM2.

**Supplementary Information:**

The online version contains supplementary material available at 10.1186/s12950-021-00286-4.

## Introduction

Chronic obstructive pulmonary disease (COPD) is the third leading cause of death globally, with cigarette smoking and exposure to smoke from biomass burning the leading risk factors for disease development [1]. Although there have been some improvements in COPD management in recent decades the therapies are restricted to symptom reduction and there is a need for more investigation into novel molecular targets of this disease. One of the pathologies of COPD is persistent airway inflammation even after cessation of smoking, which is destructive to the distal airways [1]. We have established that alveolar macrophages in COPD patients and exposure to cigarette smoke display defective phagocytic functions [2, 3] that potentially lead to increased number of uncleared apoptotic cells and secondary necrosis in contribution to self-perpetuation of inflammation [4]. Furthermore, defective phagocytosis of microbes favours survival and growth of opportunistic pathogens that are often associated with flares of inflammatory exacerbations [1, 5].

Inflammasomes are multi-protein complexes that act as platforms for cleavage (i.e. activation/maturation) of pro-cytokines of the IL-1 family (e.g. IL-1α, IL-1β, IL-18) [6], which are prominent inflammatory cytokines in COPD and other chronic lung diseases [7]. The inflammasomes are formed (activated) in the cytosol by protein aggregation around core protein-sentinels, and are named according to these proteins, e.g. NLRP3 (NACHT, LRR and PYD domains-containing protein 3), AIM2 (Absent In Melanoma protein 2), NLRC4 (NLR family CARD domain-containing protein 4) [6]. Most inflammasomes recruit the adapter protein ASC (Apoptosis-associated Speck-like protein containing a CARD), oligomerization of which can be detected by fluorescence of fusion constructs or immunofluorescence of endogenous protein as a readout of inflammasome activation [8]. Except for the NLRP3 inflammasome which senses a broad range of pathogen-derived and self-damage-derived danger signals, the other inflammasomes are believed to be restricted to specific danger signals [6]. The AIM2 inflammasome is activated by the inappropriate presence of double-stranded DNA (dsDNA) in the cytosolic compartment due to transport by pathogens, or leakage from damaged organelles [6]. Most inflammasome studies in COPD patients and cigarette smoke exposure models have focused on NLRP3 [9–13] but recent studies suggest involvement of the AIM2 inflammasome [14, 15].

In this study we investigate lung cells and tissues from COPD patients and cigarette smoke-exposed mice, and an ex vivo model of cigarette smoke extract-treated human alveolar macrophages to explore the hypothesis that the AIM2 inflammasome is activated in the airways of COPD patients and in response to cigarette smoke, and that this activation is associated with a nuclear-to-cytoplasmic shift of distribution of AIM2.

## Results

### AIM2 localized to both the cytoplasm and nucleus

**Fig. 1:**
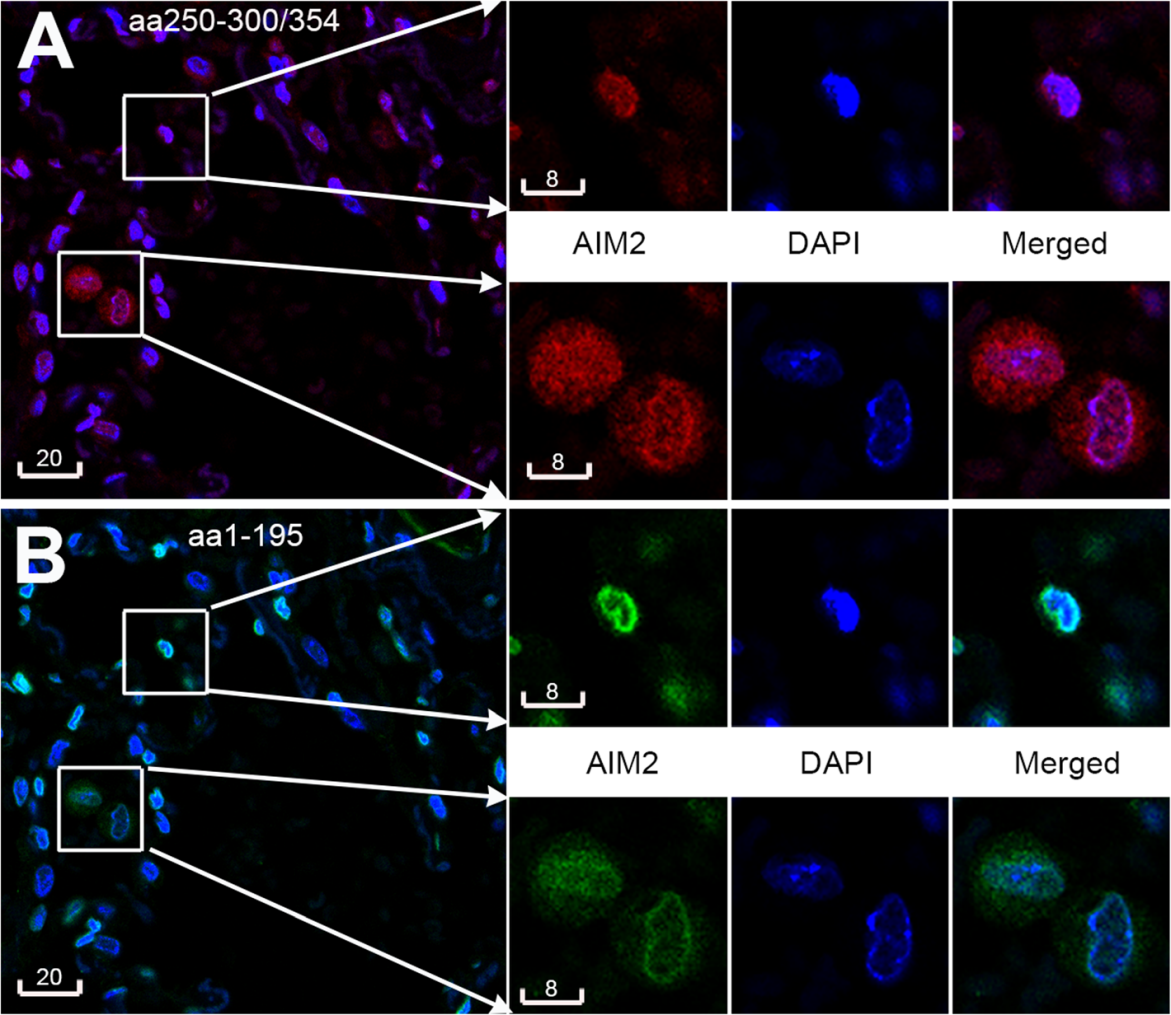
Nuclear and cytoplasmic AIM2. Representative confocal images revealing nuclear and cytoplasmic AIM2 detected with a rabbit polyclonal antibody (**a**, aa250–300/354, red by AF594) and a mouse monoclonal antibody (**b**, aa1–159, pseudogreen by AF647) in the same paraffin section of a human lung biopsies. Boxed areas are shown at magnification (right) to reveal cells having predominantly nuclear AIM2 (top box), or both nuclear and cytoplasmic AIM2 (bottom box). Blue is DAPI. Nuclear localization of AIM2 is indicated by the merged colors magenta in A and azure in B. Scale bars are in micrometers

**Fig. 2:**
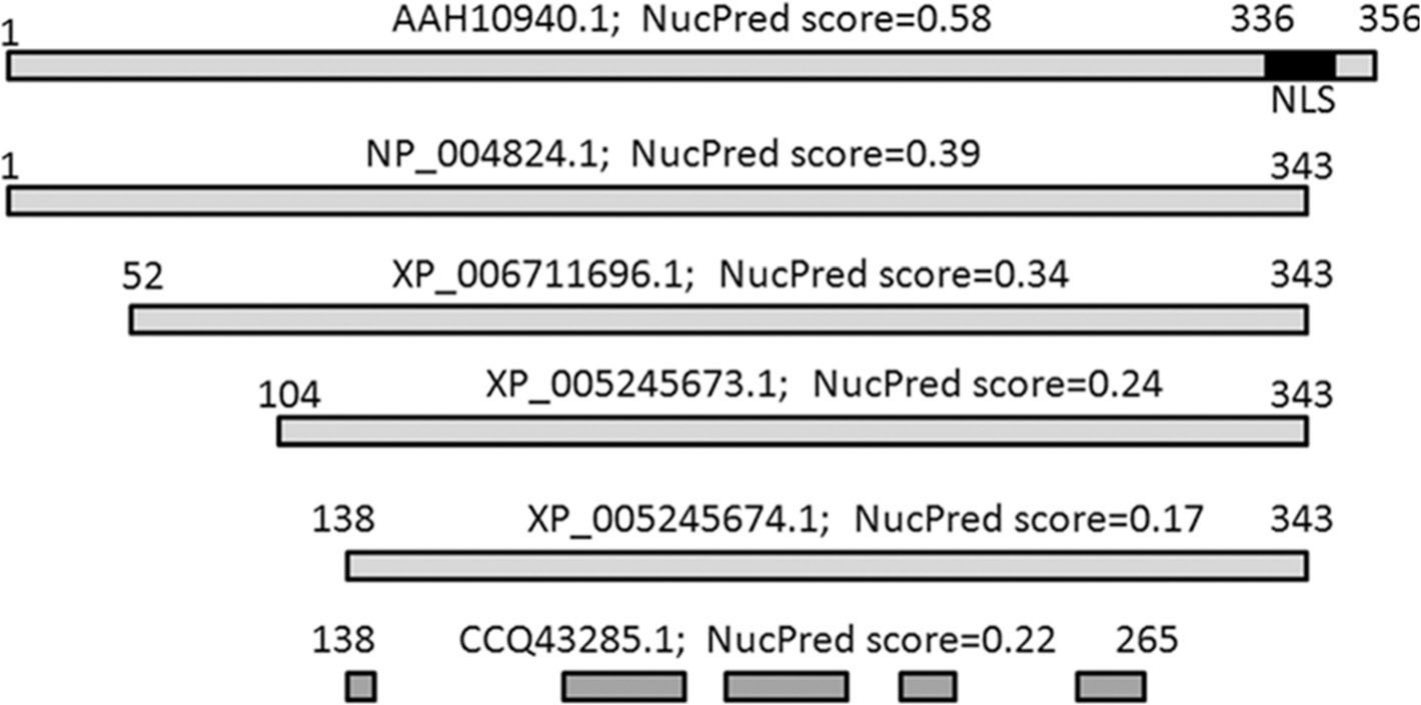
Amino acid sequence variants of human AIM2 and their Nuclear LocalizationPrediction score. NucPred: Nuclear Localization Prediction. NLS: Nuclear localization sequence signal


To best determine the cellular localization of AIM2, a panel of antibodies raised in different species against various epitope domains of human AIM2, summarized in Table S1 were compared for their immunoreactivity in paraffin and frozen sections of human lung biopsies, paraffin sections of mouse lungs, and human cell cytospins (Fig. 1, Supplementary Figs. S1 and S2). The panel of antibodies showed a similar pattern of immunoreactivity with clear staining of both cytoplasm and nucleus, independent of preservation technique, with the exception of the goat polyclonal antibody Ab4 (aa321–335) that showed conspicuous surface staining in both paraffin and frozen sections. This dual localization was detected in various cell types, including cells outside the lung (Supplementary Figs. S2, S3, S4). Cytoplasmic AIM2 immunoreactivity was present in homogenous as well as punctate patterns, the latter could be quantified as specks using uniform confocal setting of fluorescence intensity threshold and size (Figs. 1, 2). Nuclear staining was often increased at the nuclear margination (Figs. 1, 2). The nuclear immunoreactivity was confirmed by Z-analysis and line profile analysis of AIM2-DAPI colocalization in confocal sections of both human and mouse lung tissue (Supplementary Data Fig. S5, Movie S1).

**Additional file 2:**

Bio-informatic analysis was further carried out to assess nuclear localization prediction of the AIM2 protein. Ten variants of amino acid sequences of human AIM2 available from the GenBank were analyzed, confirming their difference by Nuclear Localization Prediction scores (Fig. 2). The highest Nuclear Localization Prediction score of 0.58 was obtained for the longest sequence (accession number AAH10940.1, 356 aa), while all the shorter sequences showed reduced scores between 0.39–0.1722. In keeping with this result, a predicted monopartile Nuclear Localization Signal (NLS, VIKAKKKKHRE, position 336) was present only in the full-length variant AAH10940.1 (Fig. 2).

### Increase of cytoplasmic AIM2 in airway cells of COPD patients was associated with cleavage of IL-1β

**Fig. 3:**
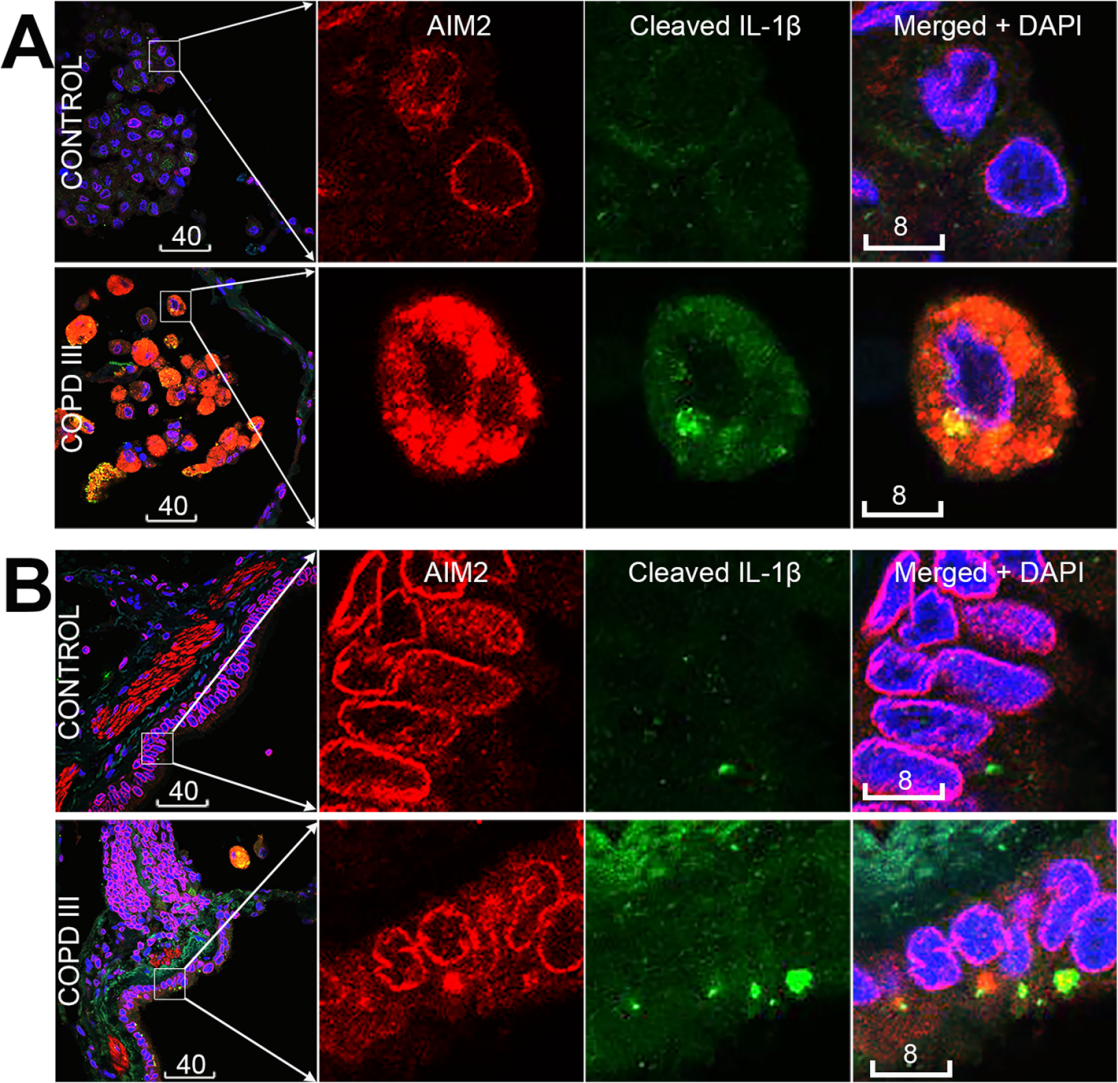
Nuclear exit of AIM2 in lungs of COPD patients was coupled with IL-1β cleavage. **a, b** Representative confocal images of alveolar macrophages (**a**) and bronchiolar epithelium (**b**) in lung biopsies from non-COPD non-smoker controls (top) and COPD-III patients (bottom). Red: AIM2 by mouse mAb #1 (aa1–195); green: cleaved IL-1β; blue: DAPI. Magenta is the merged color of red and blue, indicating nuclear localization of AIM2. Yellow/orange is the merged color of red and green, indicating colocalization of AIM2 and cleaved IL-1β (short arrows). Scale bars are in micrometers

**Fig. 4:**
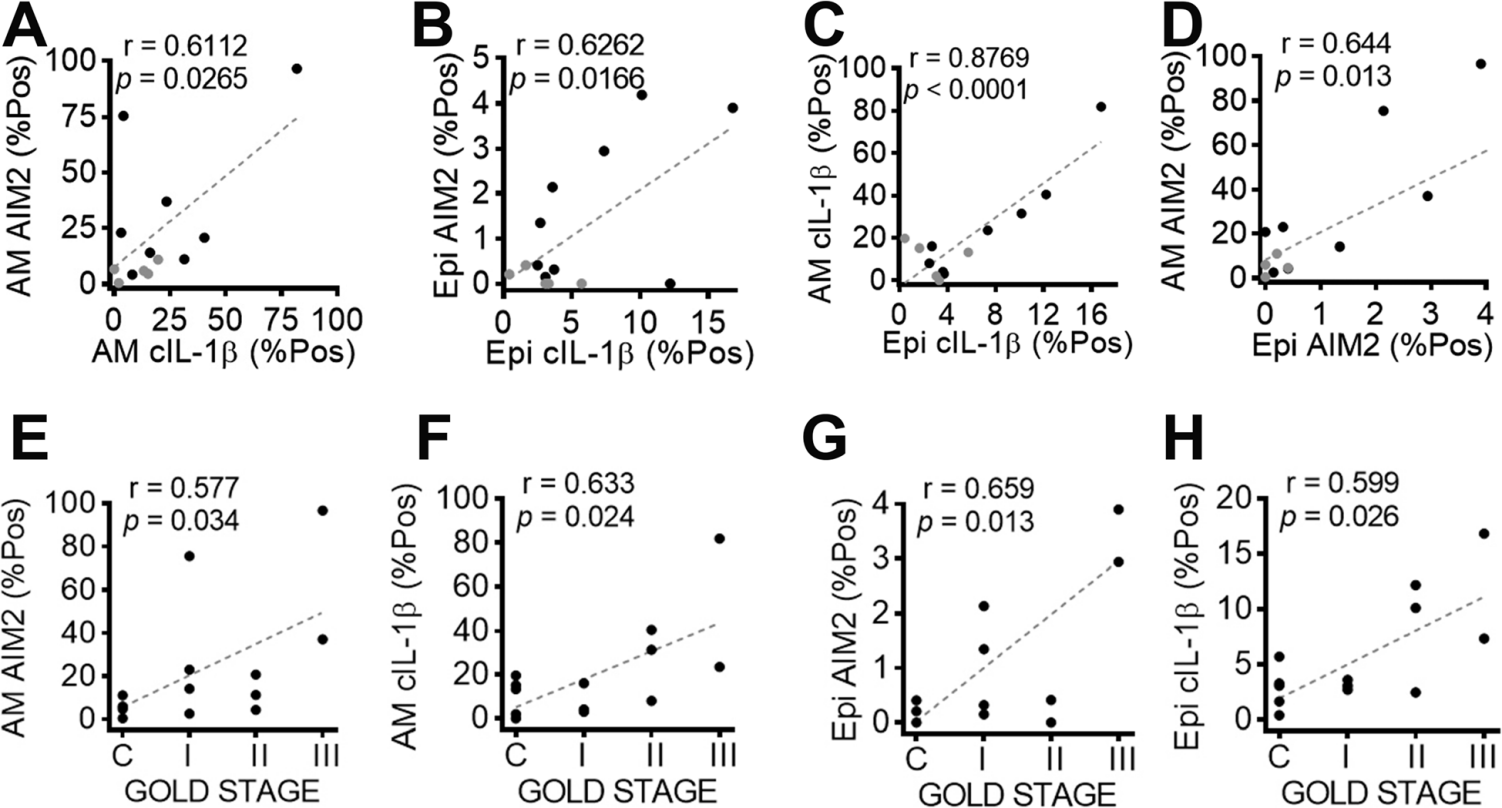
Correlation analysis of AIM2 and cleaved IL-1β specks in lung biopsies. A, B: Increase of cytoplasmic AIM2 specks directly correlated with that of cleaved IL-1β, in either alveolar macrophages (**a**) or bronchiolar epithelium (**b**). **c**, **d** Positive correlations of AIM2 and cleaved IL-1β specks between alveolar macrophages and bronchiolar epithelium. Black dots in **a**-**d** represent data sets obtained from COPD patients; grey dots: non-COPD non-smoker controls. **e** - **h**: Levels of AIM2 and cleaved IL-1β specks in each cell type positively correlated with GOLD stages. COPD, *n* = 9; non-COPD non-smoker controls, *n* = 5

A panel of 14 paraffin embedded lung biopsies taken from non-tumour adjacent tissue (as assessed by a trained pathologist) of patients undergoing lobectomy for their cancers (9 having COPD, 5 non-COPD controls, Table 1) was available for examination of protein expression and subcellular localisation of AIM2/cleaved IL-1β, and quantitative analysis of their specks. As shown above, AIM2 immunoreactivity was localized to both the cytoplasm and the nucleus in alveolar macrophages and bronchiolar epithelial cells. Cytoplasmic specks of AIM2 were co-localized and positively correlated with cleaved IL-1β (Figs. 3, 4). Importantly quantitative measurement of AIM2/ cleaved IL-1β showed significant positive correlations with GOLD stages in both alveolar macrophages and bronchiolar epithelium (Fig. 4). Speck analysis revealed also a significant positive correlation between the two cell types, though the bulk of increase observed in the former cell type (Fig. 4).

**Table 1:**
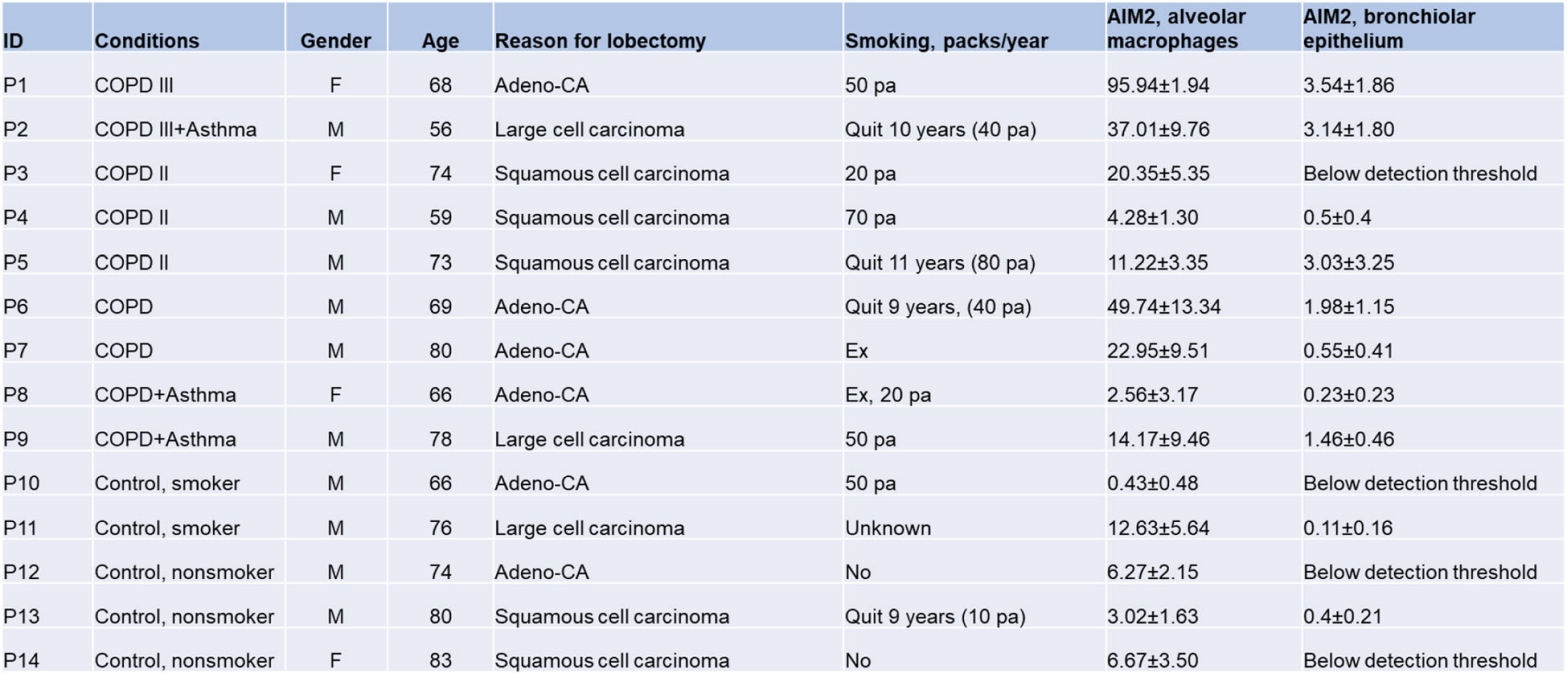
List of lobectomy patients tissue donors in the study

The results obtained from multiple labelling of paraffin sections were confirmed by analysis of cryosections of COPD (*n* = 2) and non-COPD (n = 2) lungs (Supplementary Online Fig. S6).

**Fig. 5:**
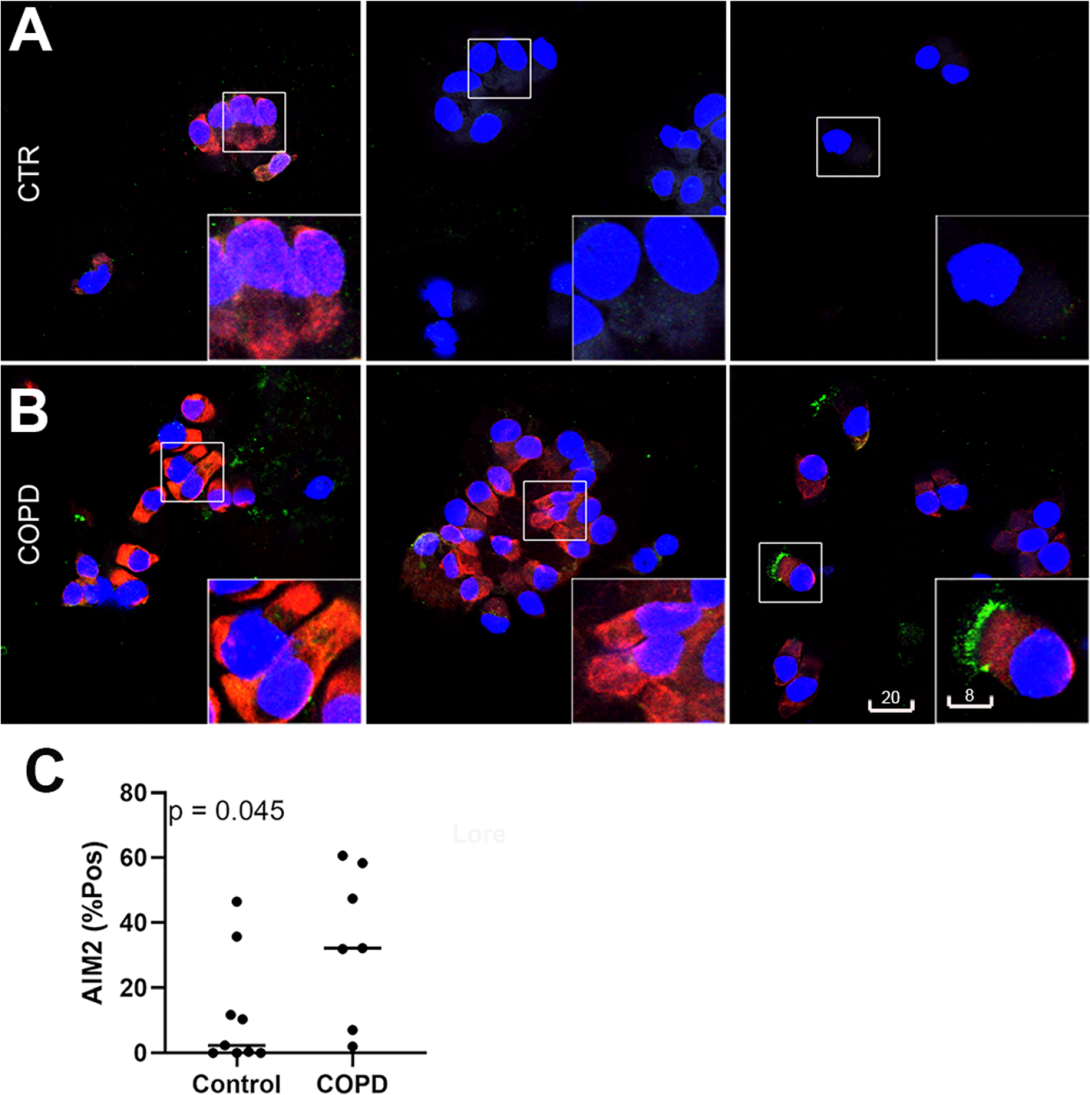
AIM2 expression and localization in airway epithelial cells obtained from COPD patients and non-COPD non-smoker controls by upper airway brushing. **a**, **b**: Representative confocal images of AIM2 (red, Ab #1, aa250–300/354) and cleaved IL-1β (green) in cells from three different COPD patients (b) vs. three non-COPD non-smoker controls (**a**). Blue: DAPI. Scale bars in **b** are in micrometers and applied for the whole panel. C: Quantitation of bright AIM2 positive cells from COPD (*n* = 7) vs. non-COPD non-smoker control (n = 9)

To confirm and further investigate the presence of AIM2 inflammasome in epithelial cells, cytospins of bronchial epithelial cells obtained via bronchoscopy-assisted brushing from a panel of 16 donors (7 COPD patients and 9 healthy controls) were examined for expression/localization of AIM2 and cleaved IL-1β. While AIM2 immunofluorescence in epithelial cells from healthy controls was dull (Fig. 5a, middle and right panels) or moderate (5A, left panel), there was a bright cytoplasmic staining for AIM2 in epithelial cells from COPD patients, often associated with specks of cleaved IL-1β (Fig. 5b), which was hardly detected in control. In contrast to tissue sections and primary cultures of alveolar macrophages, cytospins of cells freshly obtained via brushing contained a high proportion of cell debris which prevented quantitative particle analysis of fluorescence specks. Percentage of cells having high MFI were counted instead, showing a significant increase of AIM2 in COPD donors vs. control (*p* = 0.045; Fig. 5c).

### Cigarette smoke extract induced AIM2 inflammasome activation in human alveolar macrophages

**Fig. 6:**
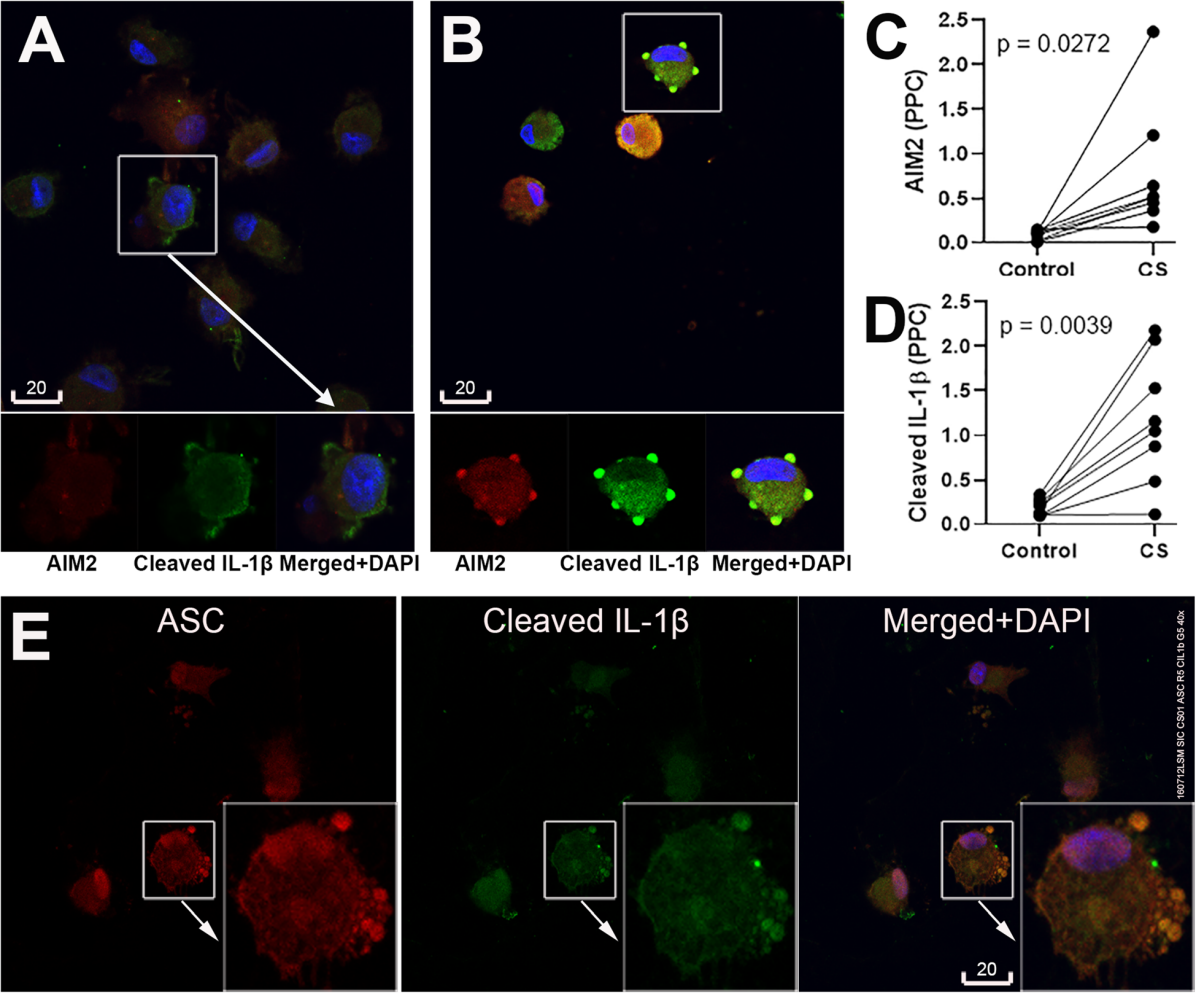
Cigarette smoke extract (CS) induced AIM2 inflammasome activation in non-COPD non-smoker control BAL derived alveolar macrophages. **a**, **b** Representative confocal images of AIM2 (red, Ab #1, aa250–300/354) and cleaved IL-1β (green) in alveolar macrophages exposed to cigarette smoke extract (B), vs. vehicle control (**a**). **c**, **d** Quantitation of AIM2 and cleaved IL-1β specks (PPC, number of particles per cell) in cells treated with cigarette smoke extract (CSE) vs. vehicle control for paired analysis (*n* = 8). **e** Colocalization of cleaved IL-1β with ASC in CSE-treated alveolar macrophages

Our observations in lobectomy biopsies indicate alveolar macrophages are the major cell type for the AIM2 inflammasome activation in the COPD airway. Therefore, BAL-derived alveolar macrophages obtained from 8 healthy non-smoker donors were further studied. The macrophages in primary cultures expressed low baseline levels of immunoreactivities for both AIM2 and cleaved IL-1β, followed by a significant induction of AIM2 and cleaved IL-1β immunofluorescence specks in response to exposure to cigarette smoke extract (Fig. 6). Similar to the in vivo situation, AIM2 and cleaved IL-1β specks were colocalized near the cell surface but also in the extracellular space (Fig. 6 and Supplementary Data Fig. S7). To further support the presence of functional inflammasomes in macrophages, co-staining with ASC was investigated, revealing increased ASC staining colocalised with cleaved IL-1β-positive particles (Fig. 6).

### AIM2 nuclear-to-cytoplasmic exit in mouse model of chronic exposure to cigarette smoke

**Fig. 7:**
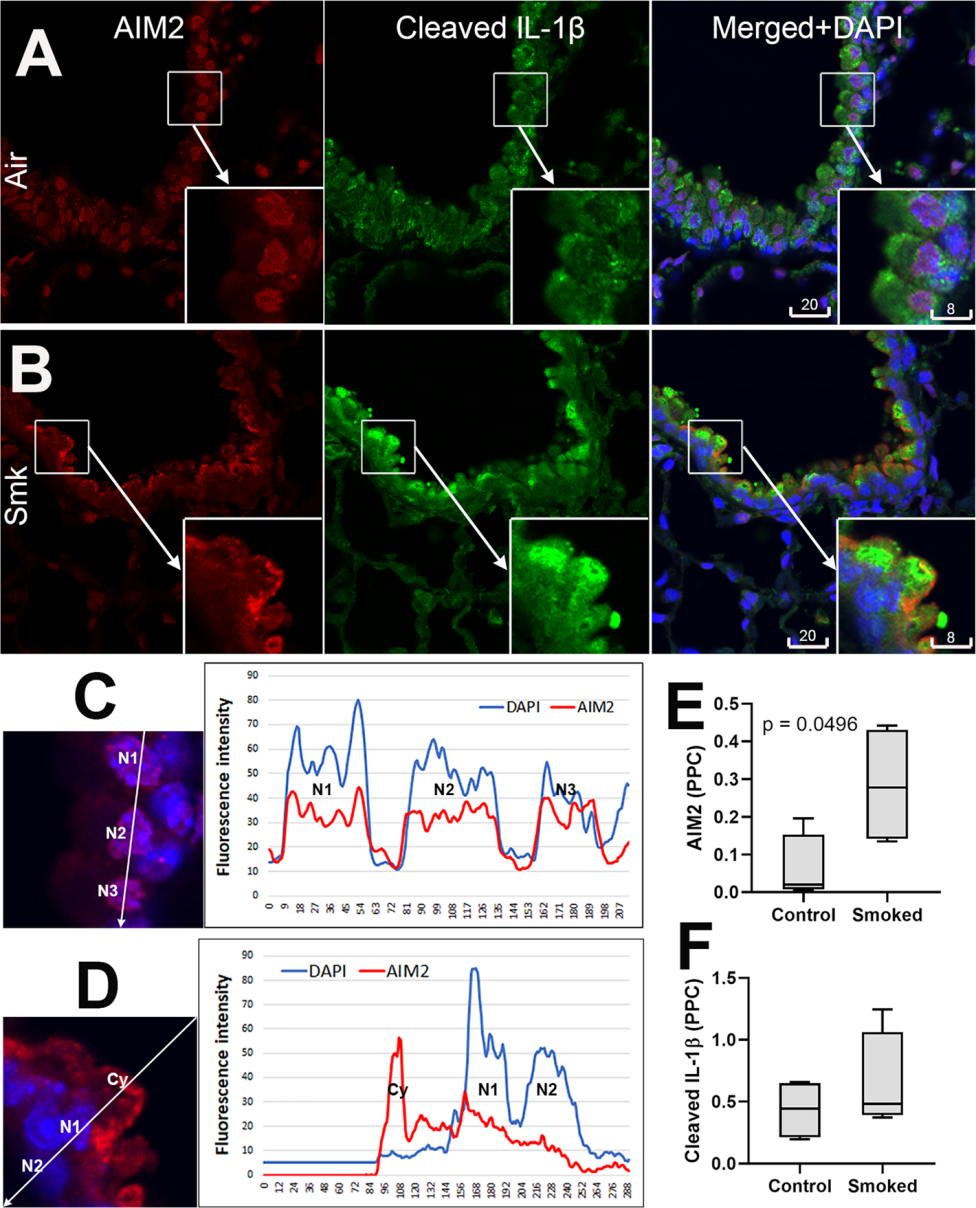
Nuclear-to-cytoplasmic translocation of AIM2 and increased cleaved IL-1β in bronchiolar epithelium of cigarette smoke-exposed mice. **a**, **b** Representative confocal images of AIM2 (red, Ab #1, aa250–300/354) and cleaved IL-1β (green) in bronchioles of mice exposed to cigarette smoke for 24 week (**b**), vs. sham control (**a**). **c**, **d** Line profile analysis revealing nuclear localization of AIM2 in control mice (**c**) and cytoplasmic in smoked mice (**d**). Pixels intensities measured along the line (inset) were plotted in y-axis, vs. their localization in x-axis. **e**, **f** Quantitation of AIM2 cytoplasmic specks and cleaved IL-1β in mouse bronchioles, cigarette smoke-exposed (*n* = 4) vs. sham control (n = 4)

We next tested if cigarette smoke, the major risk factor for COPD, can reproduce the characteristic AIM2 patterns of subcellular localization in experimental models. Lung tissue of mice chronically exposed to cigarette smoke for 24 weeks were examined for AIM2 subcellular localization, and immunoreactivity for cleaved IL-1β as indicator of a functionally active inflammasome. The shift in AIM2 subcellular distribution was found most remarkable in the epithelial cell type, but not alveolar macrophages. While bronchiolar epithelium of control mice revealed predominant nuclear localization of AIM2 immunoreactivity and low levels of immunoreactivity for cleaved IL-1β, a distinctive nuclear-to-apical translocation of AIM2 was shown in smoked mice, associated often with an increase in cleaved IL-1β. Quantitative analysis in small numbers of animals (*n* = 4 per group) confirmed that cigarette smoke exposure induced significant increase of cytoplasmic AIM2. Change in cleaved IL-1β was however not statistically significant (Fig. 7).

### AIM2 nuclear-to-cytoplasmic exit in cigarette smoke extract-stimulated HBEC30KT epithelial cell line

**Fig. 8:**
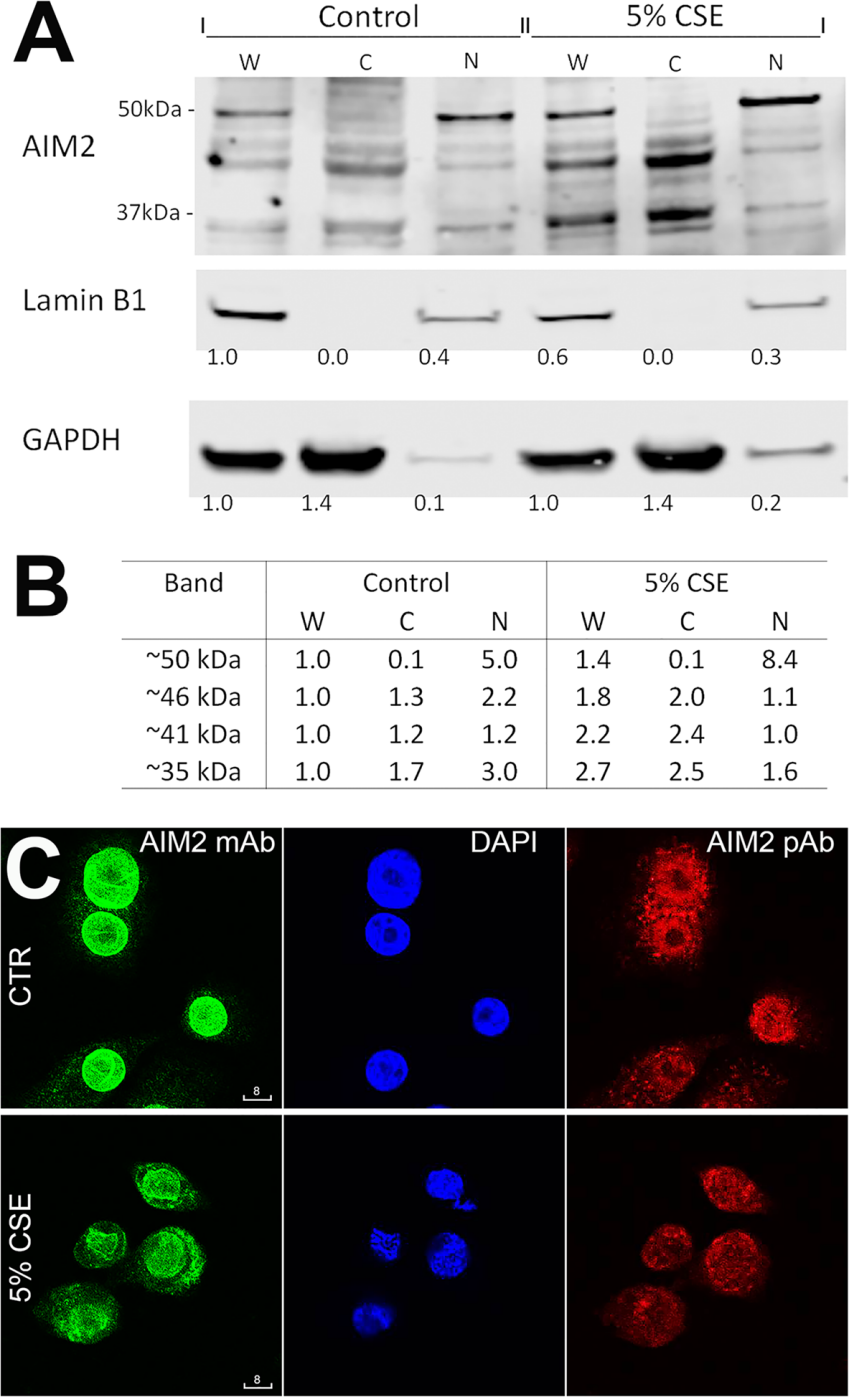
Nuclear-to-cytoplasmic redistribution of AIM2 in cigarette smoke extract- (CSE-) stimulated HBEC30KT cells. **a**: Representative Western blots analysis of AIM2 in whole cell lysate(W), cytoplasmic (**c**) and nuclear fractions (N) (*n* = 2). **b** Comparison of band abundance between CSE-stimulated and control. Band abundance was normalized to the corresponding band in the whole cell lysate and adjusted to GAPDH (cytoplasmic) or Lamin B1 (nuclear) expression. **c** Representative confocal images of HBEC30KT, control vs. CSE-stimulated (*n* = 2). Green: mouse mAb (Ab5); Red: rabbit pAb (Ab1). Blue: DAPI. Scale bars are in micrometers

Subcellular localization of AIM2 was further investigated by cell fractionation followed by immunoblotting, using the cell line HBEC30KT derived from human bronchial epithelium. The purity of the nuclear and cytoplasmic fractions was confirmed by single bands of Lamin B1 in the former, and GADPH in the latter. While immunoblotting of the total cell lysate detected multiple bands for AIM2, the lower molecular weight bands (~ 35; ~ 41 and ~ 46 kDa) were more abundant in the cytoplasmic fraction, but the higher molecular weight band (~ 50 kDa) was predominant in the nuclear fraction (Fig. 8).

The four above mentioned molecular weight bands of AIM2 were compared for their abundance after normalization to the corresponding bands in the whole cell lysate and adjustment based on the GAPDH (cytoplasmic) or Lamin B1 (nuclear) expression. Data obtained from cigarette smoke extract-stimulated vs. control cells indicated that while there was only a modest change in the total, cigarette smoke extract-stimulated cells showed a clear shift of AIM2 out of the nucleus (Fig. 8).

In line with data from immunoblotting analysis of cell fractions, confocal analysis demonstrated a distinctive nuclear-to-cytoplasmic redistribution of AIM2 protein, compared to vehicle control (Fig. 8). No significant increase of cleaved IL-1β was however observed.

## Discussion

In this study we report the first evidence of cell type-specific activation of AIM2 inflammasome in COPD airways. Although known as molecular platforms driving inflammation, the role of inflammasomes in COPD remains controversial. Faner et al. [9] reported that lungs of stable COPD patients showed an increased expression of the NLRP3 and IL-1β genes which correlated with decreasing lung function. During exacerbations, COPD patients showed activated caspase-1, oligomerization of ASC protein as indicator of inflammasome activation, and an increase of IL-1β protein in their sputum [9]. However, in another study by Di Stephano et al. [10], no significant activation of inflammasomes was found based on protein expression of caspase-1 and NLRP3 in bronchial mucosa and IL-1β, IL-18 in BAL of smokers and patients with mild/moderate or severe COPD compared to healthy non-smokers. An upregulation of the inflammasome inhibitory proteins NALP7 and IL-37 was detected instead [10]. In addition to human studies, findings on rodent models of exposure to cigarette smoke also showed differences in outcomes with results that support inflammasome activation [11, 12] or indicate suppression of it in contrast [13]. Here, differences might relate to the type of inflammasome(s) involved. Eltom et al. [12] reported that mice missing functional NLRP3 but not AIM2 protein were protected against cigarette smoke-induced IL-1β/IL-18 release, supporting NLRP3, but not AIM2 as the major inflammasome involved.

Recent studies reported AIM2 inflammasome pathway upregulation in peripheral blood mononuclear cells (PBMCs) of COPD patients during exacerbations but not during stable periods [14, 15]. Although CD14+ PBMCs showed increased AIM2 protein expression in COPD patients compared to non-COPD smokers and healthy subjects [14], stimulation of PBMCs with poly-dA:dT as surrogate dsDNA was able to induce IL-1α release only when cells were obtained from exacerbated but not stable COPD patients [15]. The AIM2 inflammasome activation in this model triggered production of detrimental TGF-β [14] and could not be alleviated with dexamethasone [15]. Although these studies point out a possible inflammatory mechanism contributing to the severity of the disease, whether the AIM2 inflammasome is activated in COPD airways and independently of exacerbation periods was not known.

The discrepancies between our and other studies could be caused by either (i) varying degrees of COPD severity, and/or (ii) the cell types available for analysis, and/or (iii) the methods used for the detection of inflammasome activation. First, our data reveals a positive correlation between readouts of the airway AIM2 inflammasome activation and GOLD stages, which is in line with recent data indicating AIM2 inflammasome activation in peripheral blood during exacerbations flares [14, 15]. Second, although in this study inflammasome activation was shown not to be limited to the myeloid lineage, with epithelial cells clearly showing cleavage of IL-1β and cytoplasmic AIM2 specks correlated with GOLD stages, numbers of ‘positive’ epithelial cells remained an order lower than in alveolar macrophages. Multifluorescence confocal microscopy can detect those cells but their low numbers might be below the threshold of detection by other methods. Third, as inflammasome activation is a process of protein aggregation, measurement of mRNA or protein-precursors instead of active forms (cleaved or oligomerized) may not necessarily reflect inflammasome activation but inflammasome priming. In this and previous studies [16, 17] we use the comparison of specks versus homogenous fluorescence which reflects the presence of oligomeric versus monomeric states of inflammasome proteins. This approach is well recognised for detection of inflammasome activation by confocal microscopy [8, 18]. With limitations related to (i) descriptive and correlative rather than causative findings, (ii) small numbers of lung tissue biopsies and (iii) using confocal speck analysis as single readout of inflammasome activation, we are careful with conclusions. Nevertheless, the data presented here, in combination with recent results from analysis of blood cells [14, 15] support the hypothesis that the AIM2 inflammasome can be considered as a potential therapeutic target in severe stages of COPD.

Results from the experimental models in this study lend further support of the AIM2 inflammasome activation in response to cigarette smoke exposure. In the ex vivo model of alveolar macrophages exposed to cigarette smoke extract, enhanced staining of ASC and AIM2 specks co-localized with cleaved IL-1β-positive foci clearly supports inflammasome activation. The mouse model of chronic exposure to cigarette smoke, and the in vitro model of short-term exposure of human cell line HBEC30KT to cigarette smoke extract suggest however, that response of the epithelial cell type to cigarette smoke exposure may not necessarily include a clear evidence of inflammasome activation. However, in both models, the epithelial cell type responded to cigarette smoke with a shift of AIM2 distribution from the nucleus to the cytoplasm, which apparently constitutes a necessary step of priming to inflammasome activation, as that was seen in the COPD airway.

The AIM2 protein belongs to the family of p200/HIN-200 proteins, most of which are nuclear residents, in line with the finding of a consensus bi-partile NLS near the N-terminus of their sequences [19, 20]. In contrast to the majority of this family, the AIM2 protein has been considered a primarily cytoplasmic resident, supported by the absence of the consensus NLS described above [20]. Data presented in this study indicates that the AIM2 protein can be either a nuclear or cytoplasmic resident. First, our bio-informatic analysis showed that in difference from all other variants of AIM2, the largest variant (AAH10940.1, 356 aa, MW 41 kDa) contains a mono-partile NLS at the C-terminus, having accordingly a predicted nuclear localization score considerably higher than all other variants, which are all truncated from the C-terminus. Second, results from cell fractionation experiment indicate that while the cytoplasmic fraction contained multiple bands of lower molecular weight AIM2, the nucleus is enriched with a higher molecular weight AIM2. Last, but not least, confocal analysis indicates a great variation of nuclear-to-cytoplasmic distribution of AIM2, apparently depending on cell types and their state of activity, but co-localized AIM2/cleaved IL-1β fluorescent speck foci were exclusively found only in the cytoplasm and not the nucleus. These multiple lines of evidences support a hypothetic redistribution of AIM2 between the nucleus and cytoplasm, which raises intriguing questions regarding how the cell avoids inflammasome activation inside the nucleus in presence of genomic double-stranded DNA, and warrants further mechanistic studies to investigate possible roles of alternate mRNA splicing, or open frame reading, or post translation modifications of AIM2 in regulation of the protein trafficking and the inflammasome activation.

In conclusion, our results support a hypothesis that the AIM2 inflammasome is activated in the airway of COPD patients and in response to exposure to cigarette smoke. The AIM2 inflammasome activation is correlated with the disease severity and coupled with the protein redistribution between the nucleus and cytoplasm, offering a possible therapeutic target.

## Materials and methods

### Human tissues and cells

Protocols were approved by human and animal ethics committees of the Royal Adelaide Hospital. Banked paraffin and frozen lung biopsies from our previous study of COPD and non-COPD control patients who were undergoing lobectomies for management of lung cancer were studied, their collection and preparation as described [21]. COPD status was assessed according to GOLD criteria. Bronchoscopy sampling of human alveolar macrophages and bronchial epithelial cells from COPD patients and non-COPD controls was conducted as previously described [22, 23]. Alveolar macrophages were re-suspended in RPMI 1640, supplemented with 10% FCS and 1% wt/vol penicillin/gentamicin at a concentration of 6 × 104 cells/mL and seeded at 30,000 cells per 0.5 cm2 in eight-well chamber slides and alveolar macrophages purified by attachment for 1 h. Alveolar macrophages were cultured for further 24 h in presence of 10% cigarette smoke extract or vehicle control as described [3, 24]. For quantitative analysis of cytoplasmic AIM2 specks and IL-1β cleavage in paraffin sections of lobectomy biopsies, patients having COPD defined by the GOLD criteria (*n* = 9), and non-COPD patients (*n* = 5) were included. Patients having asthma but no COPD were excluded; details of the included subjects are presented in Table 1.

### Murine tissues

A mouse model of chronic exposure to cigarette smoke was carried out as described according to protocols approved by the institutional Ethics Committee [25, 26]. Briefly, female BALB/c mice (8-10 weeks of age) were exposed to three filtered cigarettes over a 1 h period, three times per day, 5 days per week, for the total of 24 weeks. At the end of experiment, mouse lungs were inflation fixed with neutral buffered formalin (NBF), dissected, fixed in NBF for 24 h and processed into paraffin blocks.

### Immunofluorescence and antibodies

Indirect (two step) immunofluorescence of inflammasome proteins in tissue [17, 27] and cells [16] was performed according to previously published protocols. Five commercial antibodies (Ab1–5) raised in different species against different immunogenic peptides derived from human AIM2 were used in the study and listed in Table S1. Other primary antibodies, directed against human cleaved IL-1β (goat polyclonal), mouse cleaved IL-1β (goat polyclonal), and anti-ASC (mouse monoclonal) were from Santa Cruz. The specificity of the antibodies to cleaved human and cleaved mouse IL-1β was confirmed by pre-incubation and blocking specific labelling with the immunogen peptides. The secondary antibodies were AF488, AF594 and AF647-conjugated donkey serum IgG F (ab’)2 fragments, obtained from Jackson ImmunoResearch (West Grove, PA, USA).

### Multifluorescence quantitative confocal microscopy

Multifluorescence confocal microscopy for quantitative analysis [28] was described in details in [17], using a LSM700 confocal system (CarlZeiss Australia, NorthRyde, NSW, Australia). Inflammasome activation was detected by speck foci of AIM2 and cleaved IL-1β as previously described [16, 17]. Briefly, using the particle analysis function of the ImageJ software (NIH, Bethesda, MA, USA), the lower threshold of fluorescence intensity was set uniformly high to gate in only bright particles at uniformly selected sizes, which were then counted and results normalized to the cell numbers counted separately by counting DAPI-stained nuclei.

### Bio-informatic analysis

By searching in Protein PubMed, using keywords “AIM2” and “*Homo sapiens*”, 10 variants of human AIM2 amino acid sequences previously deposited in the Gene Bank were obtained. Variant sequences were aligned as in Fig. 2, using a tool available at http://www.ebi.ac.uk/Tools/msa/clustalo/. The sequences were then submitted to online softwares for Nuclear Localization Prediction score [29] and prediction of Nuclear Localization Signal [30].

### HBEC30KT cell culture, cell fractionation and Western blot analysis

The previously characterised Cdk4 (cyclin-dependent kinase 4) and hTERT (human telomerase reverse transcriptase)-immortalized human bronchial epithelial cell line HBEC30KT were a gift from Dr. John Minna and were maintained in Keratinocyte-SFM (Thermo Fisher Scientific, Scoresby VIC) containing 50 mg/mL bovine pituitary extract and 5 ng/mL epithelial growth factor, as previously described [31, 32]. HBEC30KT cells were treated with 5% CSE or vehicle control for 24 h prior to being scraped directly into ice-cold nuclear isolation buffer (NIB: 10 mM HEPES, 10 mM potassium, 0.1 mM EDTA). Cells were disrupted by 10 passes through a 26.5G needle before pelleting at 800 x *g* for 10 min at 4 °C, the supernatant was collected as the cytosolic fraction, pellets were washed twice in NIB before being lysed in nuclear extraction buffer (NEB, 10 mM HEPES, 10 mM potassium, 0.1 mM EDTA, 400 mM sodium chloride, 1% v/v Igepal Ca-630) on ice for 15 min before clearing the nuclear lysate at 17,000 x *g* for 10 min at 4 °C. For whole-cell lysates, cells were lysed directly into NEB and treated as above. NIB and NEB were supplemented with 1 mM DTT, 10 mM B-glycerophosphate, 2 mM Sodium orthovanadate, 2 mM sodium fluoride, 10 mM sodium pyrophosphate and protease inhibitor cocktail (Roche, Castle Hill NSW). Lysates were quantitated using BCA assay (Thermo Fisher Scientific) and 10μg of total protein or equivalent volumes of cytosolic and nuclear lysates were loaded onto Bio-Rad (Gladesville NSW) 4-15% TGX gels before transferring to nitrocellulose. Antibodies diluted in Odyssey blocking buffer (LI-COR, NE USA) were incubated overnight at 4 °C, rabbit anti-AIM2 (1:1000, Bioss. MA USA), rabbit anti-GAPDH (1:5000, 14C10, Cell Signalling Technology, MA USA) and rabbit anti-Lamin B1 (1:10,000, PA5–86096, Thermo), before detection with goat anti-rabbit 800 (LI-COR) on an Odyssey CLX scanner (LI-COR).

### Statistical analysis

Mann-Whitney U-test was employed for differences between subgroups of patients, paired T-test for difference induced by cigarette smoke extract in primary cell cultures and correlation analysis by Pearson’s test. All analyses were undertaken using Prism software.

## Supplementary Information

**Additional file 1: Table S1.** AIM2 antibodies used in the study and their immunoreactivities. **Figure S1.** The majority of tested AIM2 antibodies displayed both nuclear and cytoplasmic immunoreactivity. **Figure S2.** Nuclear and cytoplasmic localization of AIM2 expressed by different cell types in frozen sections of human lung biopsies. Shown is a representative confocal image of the same section labeled with a mouse monoclonal (Ab5, aa1–195, red) and a rabbit polyclonal (Ab1, aa250–300/354, green) antibodies, revealing similar nuclear and cytoplasmic patterns of subcellular localization. AM: alveolar macrophages, Avl: alveolar wall cells, Vs: microvessel. The inset is magnification of the boxed area, revealing a macrophage and an endothelial cell. Blue is DAPI. Merged colors magenta (left) and azure (right) indicate nuclear localization of AIM2. Scale bars are in micrometers. **Figure S3.** Immunolocalization of AIM2 (red) and cleaved IL-1β (green) in a representative turbinate biopsies. Arrowhead: strong AIM2 nuclear signal in surface epithelium. Arrow: reduced nuclear AIM2 in glandular epithelium associated with increased cleaved IL-1β (green). **Figure S4.** Nuclear AIM2 in undifferentiated THP-1 monocytes. Ab #2 (aa232–309). Top: Microphoto taken with conventional immunofluorescence microscope. Bottom: Line profile analysis showing nuclear exit of AIM2 in the second right cell. **Figure S5.** Line profile analysis of subcellular localization of AIM2 and cleaved IL-1β in a COPD II patient’s airway. Shown are 4 nuclei, having relatively high AIM2 which (N1) or reduced AIM2 (N2,3,4). A cytoplasmic cluster of AIM2 is colocalized with cleaved IL-1β (Cy). Lines in the graph are colored in red for AIM2, green for cleaved IL-1β, and blue for DAPI. **Figure S6.** Subcellular localization of AIM2 in frozen sections of lung biopsies from non-COPD control patients (A and B) and from COPD patients (C and D). AIM2 (red) was labeled with a mouse monoclonal antibody (aa1–195). Blue is DAPI. Scale bars are in micrometers. Additonal file Representative confocal image of cigarette smoke extract-treated alveolar macrophages, showing intracellular (short arrows) and extracellular (arrowheads) of cleaved IL-1β particles colocalized with AIM2. Blue is DAPI. Scale bars are in micrometers.

## Data Availability

All data and materials are available in the main body of the manuscript and indicated links to the Online Supplementary Data.

## References

[1] Barnes PG, Burney PGJ, Silverman EK, Celli BR, Vestbo J, Wedzicha JA (2015). Chronic obstructive pulmonary disease. Nat Rev Dis Primers.

[2] Hodge S, Hodge G, Scicchitano R, Reynolds PN, Holmes M (2003). Alveolar macrophages from subjects with COPD are deficient in their ability to phagocytose apoptotic airway epithelial cells. Immunol Cell Biol.

[3] Hodge S, Hodge G, Ahern J, Jersmann H, Holmes M, Reynolds PN (2007). Smoking alters alveolar macrophage recognition and phagocytic ability: implications in chronic obstructive pulmonary disease. Am J Respir Cell Mol Biol.

[4] Hodge S, Hodge G, Holmes M, Reynolds PN (2005). Increased apoptosis in the airways in COPD persists after smoking cessation. Eur Respir J.

[5] Lopez-Campos JL, Miravitlles M, de la Rosa CD, Cantón R, Soler-Cataluña JJ, Martinez-Garcia MA (2020). Current challenges in chronic bronchial infection in patients with chronic obstructive pulmonary disease. J Clin Med.

[6] Latz E, Xiao TS, Stutz A. Activation and regulation of the inflammasomes. Nat Rev Immunol. 2013;13(6). 10.1038/nri3452. 10.1038/nri3452 PMC3807999 23702978

[7] Garth J, Barnes JW, Krick S (2018). Targeting cytokines as evolving treatment strategies in chronic inflammatory airway diseases. Int J Mol Sci.

[8] Beilharz M, De Nardo LE, Franklin BS (2016). Speck formation by confocal microscopy and immunofluorescence. Methods Mol Biol.

[9] Faner R, Sobradillo P, Noguera A, Gomez C, Cruz T, López-Giraldo A, et al. The inflammasome pathway in stable COPD and acute exacerbations. ERJ Open Res. 2016;2(3):00002–2016. 10.1183/23120541.00002-2016 PMC5034597 27730204

[10] Di Stefano A, Caramori G, Barczyk A, Vicari C, Brun P, Zanini A (2014). Innate immunity but not NLRP3 inflammasome activation correlates with severity of stable COPD. Thorax..

[11] Pauwels NS, Bracke KR, Dupont LL, Van Pottelberge GR, Provoost S, Vanden Berghe T (2011). Role of IL-1α and the Nlrp3/caspase-1/IL-1β axis in cigarette smoke-induced pulmonary inflammation and COPD. Eur Respir J.

[12] Eltom S, Belvisi MG, Stevenson CS, Maher SA, Dubuis E, Fitzgerald KA (2014). Role of the inflammasome-caspase1/11-IL-1/18 axis in cigarette smoke driven airway inflammation: an insight into the pathogenesis of COPD. PLoS One.

[13] Ye P, Wang X, Ge S, Chen W, Wang W, Han X (2019). Long-term cigarette smoking suppresses NLRP3 inflammasome activation in oral mucosal epithelium and attenuates host defense against Candida albicans in a rat model. Biomed Pharmacother.

[14] Colarusso C, Terlizzi M, Molino A, Imitazione P, Somma P, Rega R (2019). AIM2 inflammasome activation leads to IL-1α and TGF-β release from exacerbated chronic obstructive pulmonary disease-derived peripheral blood mononuclear cells. Front Pharmacol.

[15] Molino A, Terlizzi M, Colarusso C, Rossi A, Somma P, Saglia A (2019). AIM2/IL-1α/TGF-β Axis in PBMCs from exacerbated chronic obstructive pulmonary disease (COPD) patients is not related to COX-2-dependent inflammatory pathway. Front Physiol.

[16] Chen AC, Tran HB, Xi Y, Yerkovich ST, Baines KJ, Pizzutto SJ, et al. Multiple inflammasomes may regulate the interleukin-1-driven inflammation in protracted bacterial bronchitis. ERJ Open Res. 2018;4(1):00130-2017. 10.1183/23120541.00130-2017 PMC5868518 29594175

[17] Tran HB, Macowan MG, Abdo A, Donnelley M, Parsons D, Hodge S (2020). Enhanced inflammasome activation and reduced sphingosine-1 phosphate S1P signalling in a respiratory mucoobstructive disease model. J Inflamm.

[18] de Mattos Barbosa MG, de Andrade Silva BJ, Assis TQ, da Silva Prata RB, Ferreira H, Andrade PR (2018). Autophagy impairment is associated with increased inflammasome activation and reversal reaction development in multibacillary leprosy. Front Immunol.

[19] Briggs LJ, Johnstone RW, Elliot RM, Xiao CY, Dawson M, Trapani JA (2001). Novel properties of the protein kinase CK2-site-regulated nuclear- localization sequence of the interferon-induced nuclear factor IFI 16. Biochem J.

[20] Choubey D, Duan X, Dickerson E, Ponomareva L, Panchanathan R, Shen H (2010). Interferon-inducible p200-family proteins as novel sensors of cytoplasmic DNA: role in inflammation and autoimmunity. J Interf Cytokine Res.

[21] Cordts F, Pitson S, Tabeling C, Gibbins I, Moffat DF, Jersmann H (2011). Expression profile of the sphingosine kinase signalling system in the lung of patients with chronic obstructive pulmonary disease. Life Sci.

[22] Hodge S, Hodge G, Jersmann H, Matthews G, Ahern J, Holmes M (2008). Azithromycin improves macrophage phagocytic function and expression of mannose receptor in COPD. Am J Respir Crit Care Med.

[23] Tran HB, Jersmann H, Truong TT, Hamon R, Roscioli E, Ween M (2017). Disrupted epithelial/macrophage crosstalk via spinster homologue 2-mediated S1P signaling may drive defective macrophage phagocytic function in COPD. PLoS One.

[24] Ween M, Ahern J, Carroll A, Hodge G, Pizzutto S, Jersmann H (2016). A small volume technique to examine and compare alveolar macrophage phagocytosis of apoptotic cells and non typeable Haemophilus influenzae (NTHi). J Immunol Methods.

[25] Hodge S, Matthews G, Dean MM, Ahern J, Djukic M, Hodge G (2010). Therapeutic role for mannose-binding lectin in cigarette smoke-induced lung inflammation? Evidence from a murine model. Am J Respir Cell Mol Biol.

[26] Macowan MG, Liu H, Keller MD, Ween M, Rhys Hamon R, Tran HB, Hodge S. Interventional low-dose azithromycin attenuates cigarette smoke-induced emphysema and lung inflammation in mice. Accepted 21/3/2020 for publication at Phys Rep. 10.14814/phy2.14419 PMC7354087 32652854

[27] Tran HB, Lewis MD, Tan LW, Lester SE, Baker LM, Ng J (2012). Immunolocalization of NLRP3 Inflammasome in normal murine airway epithelium and changes following induction of ovalbumin-induced airway inflammation. J Allergy (Cairo).

[28] Trotman WE, Taatjes DJ, Bovill EG (2013). Multifluorescence confocal microscopy: application for a quantitative analysis of hemostatic proteins in human venous valves. Methods Mol Biol.

[29] Brameier M, Krings A, MacCallum RM (2007). NucPred-predicting nuclear localization of proteins. Bioinformatics..

[30] Kosugi S, Hasebe M, Tomita M, Yanagawa H (2009). Systematic identification of cell cycle-dependent yeast Nucleocytoplasmic shuttling proteins by prediction of composite motifs. Proc Natl Acad Sci U S A.

[31] Ramirez RD, Sheridan S, Luc Girard L, Sato M, Kim Y, Pollack J (2004). Immortalization of human bronchial epithelial cells in the absence of viral oncoproteins. Cancer Res.

[32] Kim HS, Mendiratta S, Kim J, Pecot CV, Larsen JE, Zubovych I (2013). Systematic identification of molecular subtype-selective vulnerabilities in non-small-cell lung cancer. Cell.

