# Supplementary material for: AIM2 nuclear exit and inflammasome activation in chronic obstructive pulmonary disease and response to cigarette smoke
Source: J Inflamm (Lond). 2021 May 22;18:19. doi: 10.1186/s12950-021-00286-4 (PMC8141226; doi:10.1186/s12950-021-00286-4)
Supplement: Supplementary file 2 — Additional file 1: Table S1. AIM2 antibodies used in the study and their immunoreactivities. Figure S1. The majority of tested AIM2 antibodies displayed both nuclear and cytoplasmic immunoreactivity. Figure S2. Nuclear and cytoplasmic localization of AIM2 expressed by different cell types in frozen sections of human lung biopsies. Shown is a representative confocal image of the same section labeled with a mouse monoclonal (Ab5, aa1–195, red) and a rabbit polyclonal (Ab1, aa250–300/354, green) antibodies, revealing similar nuclear and cytoplasmic patterns of subcellular localization. AM: alveolar macrophages, Avl: alveolar wall cells, Vs: microvessel. The inset is magnification of the boxed area, revealing a macrophage and an endothelial cell. Blue is DAPI. Merged colors magenta (left) and azure (right) indicate nuclear localization of AIM2. Scale bars are in micrometers. Figure S3. Immunolocalization of AIM2 (red) and cleaved IL-1β (green) in a representative turbinate biopsies. Arrowhead: strong AIM2 nuclear signal in surface epithelium. Arrow: reduced nuclear AIM2 in glandular epithelium associated with increased cleaved IL-1β (green). Figure S4. Nuclear AIM2 in undifferentiated THP-1 monocytes. Ab #2 (aa232–309). Top: Microphoto taken with conventional immunofluorescence microscope. Bottom: Line profile analysis showing nuclear exit of AIM2 in the second right cell. Figure S5. Line profile analysis of subcellular localization of AIM2 and cleaved IL-1β in a COPD II patient’s airway. Shown are 4 nuclei, having relatively high AIM2 which (N1) or reduced AIM2 (N2,3,4). A cytoplasmic cluster of AIM2 is colocalized with cleaved IL-1β (Cy). Lines in the graph are colored in red for AIM2, green for cleaved IL-1β, and blue for DAPI. Figure S6. Subcellular localization of AIM2 in frozen sections of lung biopsies from non-COPD control patients (A and B) and from COPD patients (C and D). AIM2 (red) was labeled with a mouse monoclonal antibody (aa1–195). Blue is DAPI. Scale b [file 12950_2021_286_MOESM1_ESM.pdf]

AIM2 NUCLEAR EXIT AND INFLAMMASOME ACTIVATION IN COPD AND RESPONSE TO CIGARETTE SMOKE

Supplementary Data

Table S1. AIM2 antibodies used in the study and their immunoreactivities

#	Species	Immunogen	Paraffin sections		Cells/frozen sections		Supplier
			**Nucleus**	**Cytoplasm**	**Nucleus**	**Cytoplasm**	
1	Rabbit pAb	aa250-300/354	Dull/Bright	Bright dots	Moderate	Moderate	Bioss
2	Rabbit pAb	aa232-309	Bright	Bright dots	Moderate	Moderate	Abcam
3	Rabbit pAb	aa93-342	None	None	Bright/dull	Moderate	LifeSpan
4	Goat pAb	aa321-335	Dull/None	Bright	Dull/None	Bright	Sigma
5	Mouse mAb	aa1-195	Dull/Bright	Rare dots	Dull/Bright	Rare dots	Santa Cruz

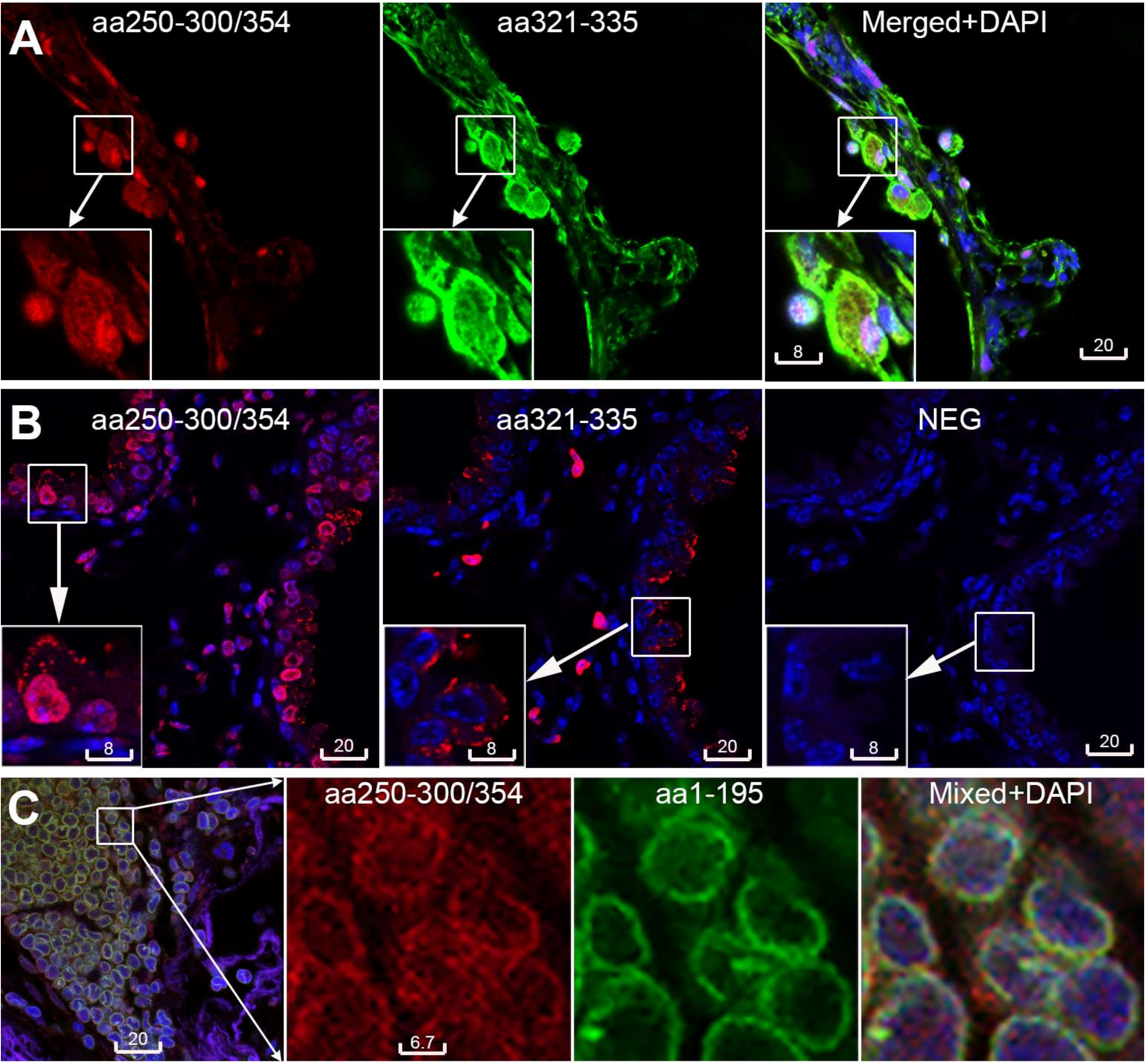

Figure S1. The majority of tested AIM2 antibodies displayed both nuclear and cytoplasmic immunoreactivity. **A:** Human frozen lung section co-labelled with Ab1 (red) and Ab4 (green). Inset is amplification of the boxed area depicting alveolar macrophages next to alveolar wall. **B:** Mouse paraffin lung section. AIM2 labeled in red by Ab1 and Ab4 in adjacent serial sections. NEG is a conjugate alone negative control. Shown are bronchiolar epithelial cells. **C:** Human lymph node in human lung, AIM2 labeled in red by a rabbit polyclonal (Ab1) and green by a mouse monoclonal (Ab5) on the same sections. Blue is DAPI. Scale bars are in micrometers. Amino acid sequences are indicated for epitope domains used for raising antibodies.

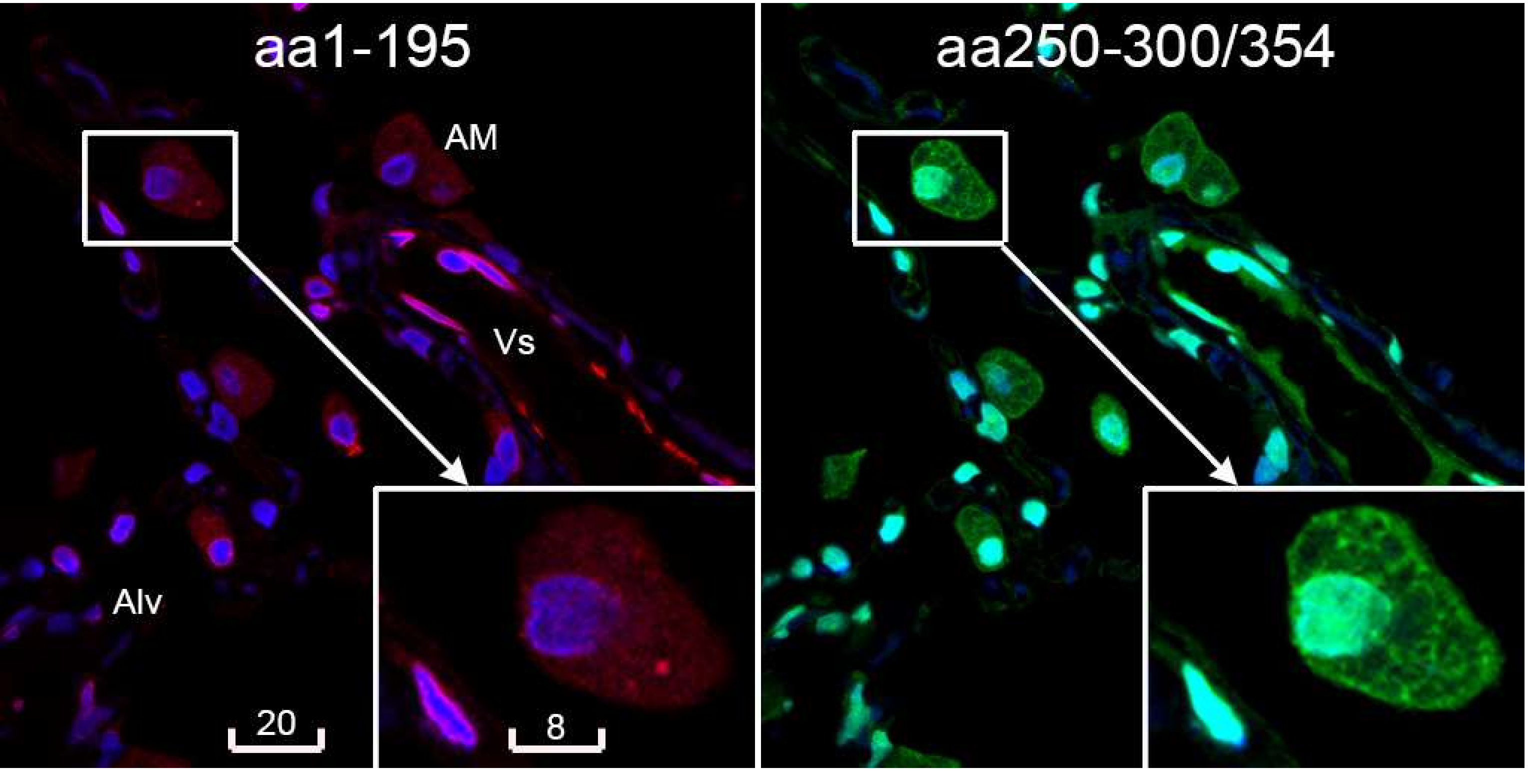

Figure S2. Nuclear and cytoplasmic localization of AIM2 expressed by different cell types in frozen sections of human lung biopsies. Shown is a representative confocal image of the same section labeled with a mouse monoclonal (Ab5, aa1-195, red) and a rabbit polyclonal (Ab1, aa250-300/354, green) antibodies, revealing similar nuclear and cytoplasmic patterns of subcellular localization. AM: alveolar macrophages, Avl: alveolar wall cells, Vs: microvessel. The inset is magnification of the boxed area, revealing a macrophage and an endothelial cell. Blue is DAPI. Merged colors magenta (left) and azure (right) indicate nuclear localization of AIM2. Scale bars are in micrometers.

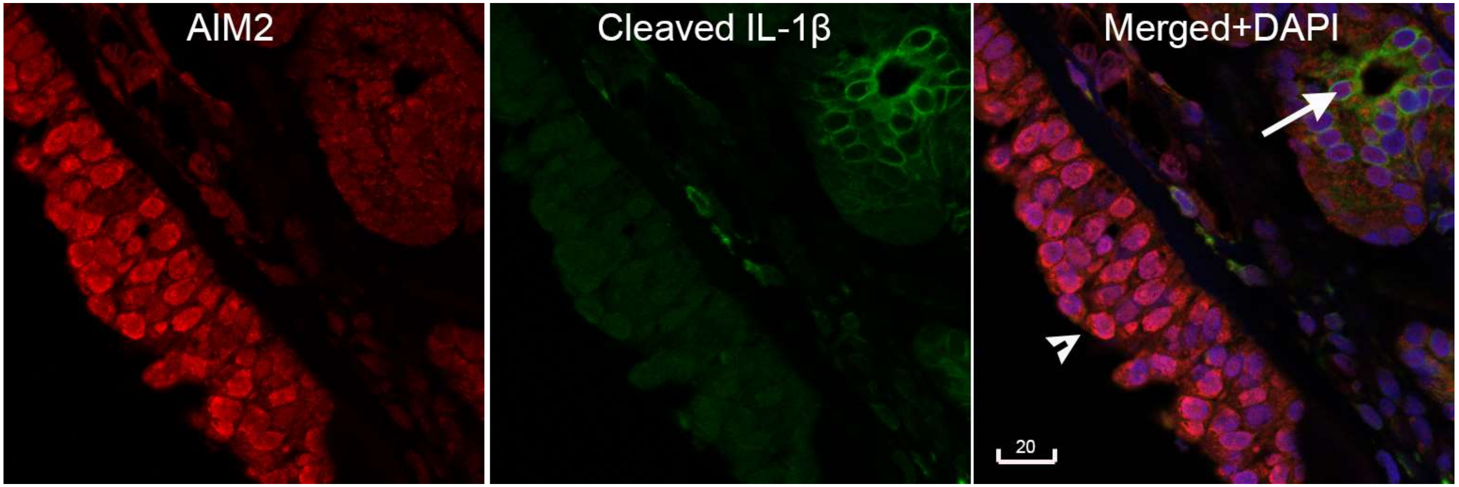

Fig S3. Immunolocalization of AIM2 (red) and cleaved IL-1β (green) in a representative turbinate biopsies. Arrowhead: strong AIM2 nuclear signal in surface epithelium. Arrow: reduced nuclear AIM2 in glandular epithelium associated with increased cleaved IL-1β (green).

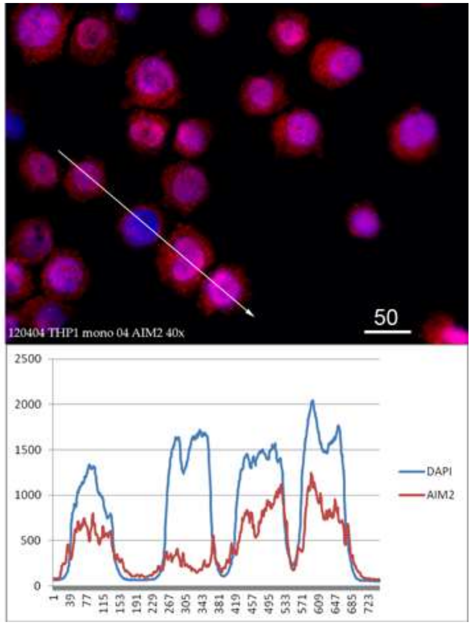

Fig S4. Nuclear AIM2 in undifferentiated THP-1 monocytes. Ab #2 (aa232-309). Top: Microphoto taken with conventional immunofluorescence microscope. Bottom: Line profile analysis showing nuclear exit of AIM2 in the second right cell.

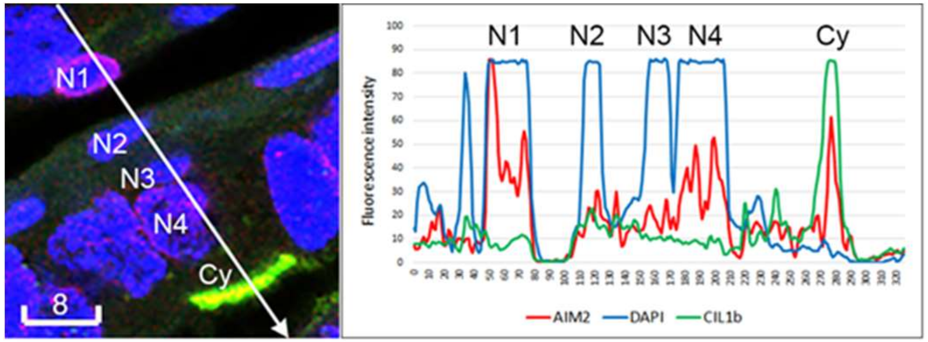

Fig S5. Line profile analysis of subcellular localization of AIM2 and cleaved IL-1β in a COPD II patient's airway. Shown are 4 nuclei, having relatively high AIM2 which (N1) or reduced AIM2 (N2,3,4). A cytoplasmic cluster of AIM2 is colocalized with cleaved IL-1β (Cy). Lines in the graph are colored in red for AIM2, green for cleaved IL-1β, and blue for DAPI

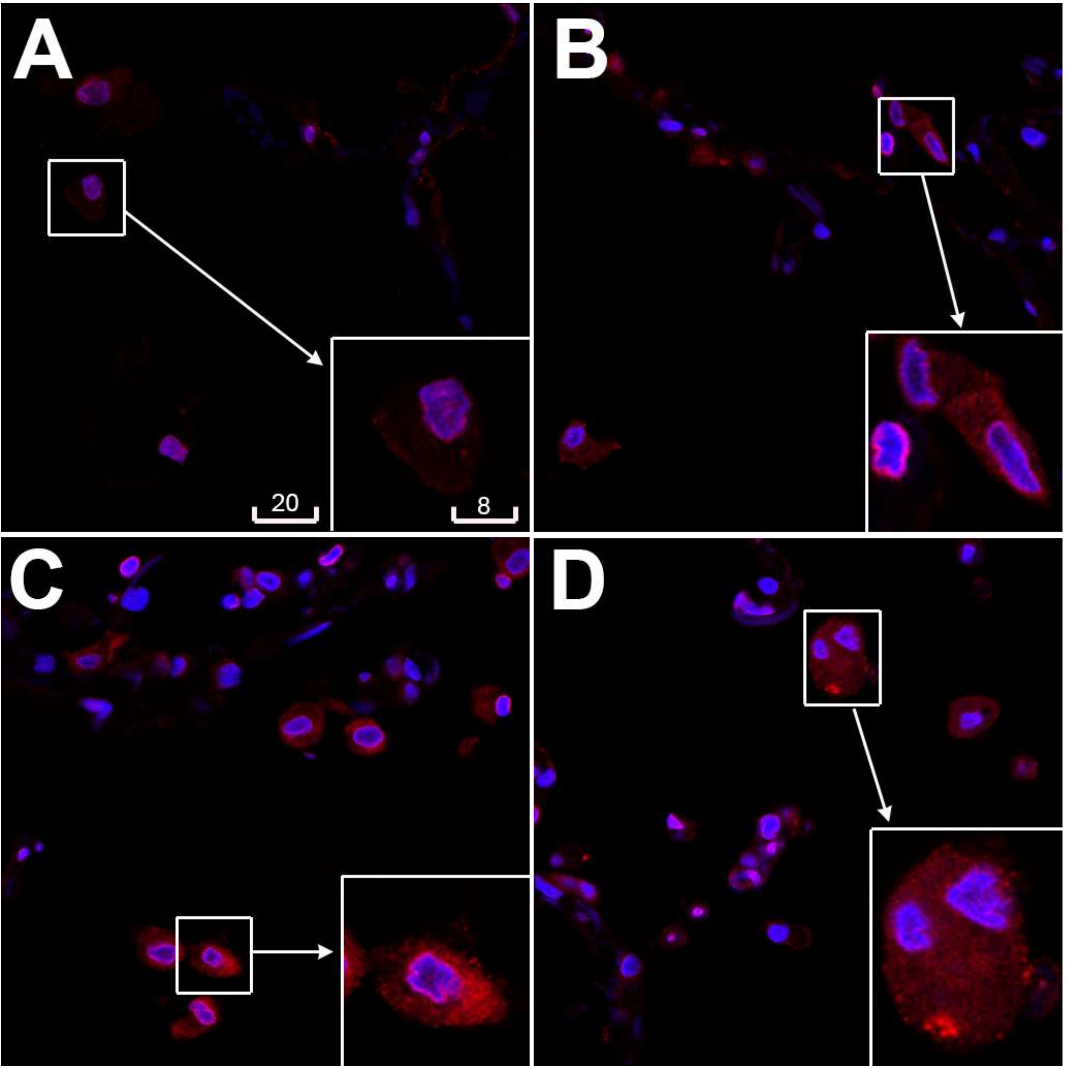

Figure S6. Subcellular localization of AIM2 in frozen sections of lung biopsies from non-COPD control patients (A and B) and from COPD patients (C and D). AIM2 (red) was labeled with a mouse monoclonal antibody (aa1-195). Blue is DAPI. Scale bars are in micrometers.

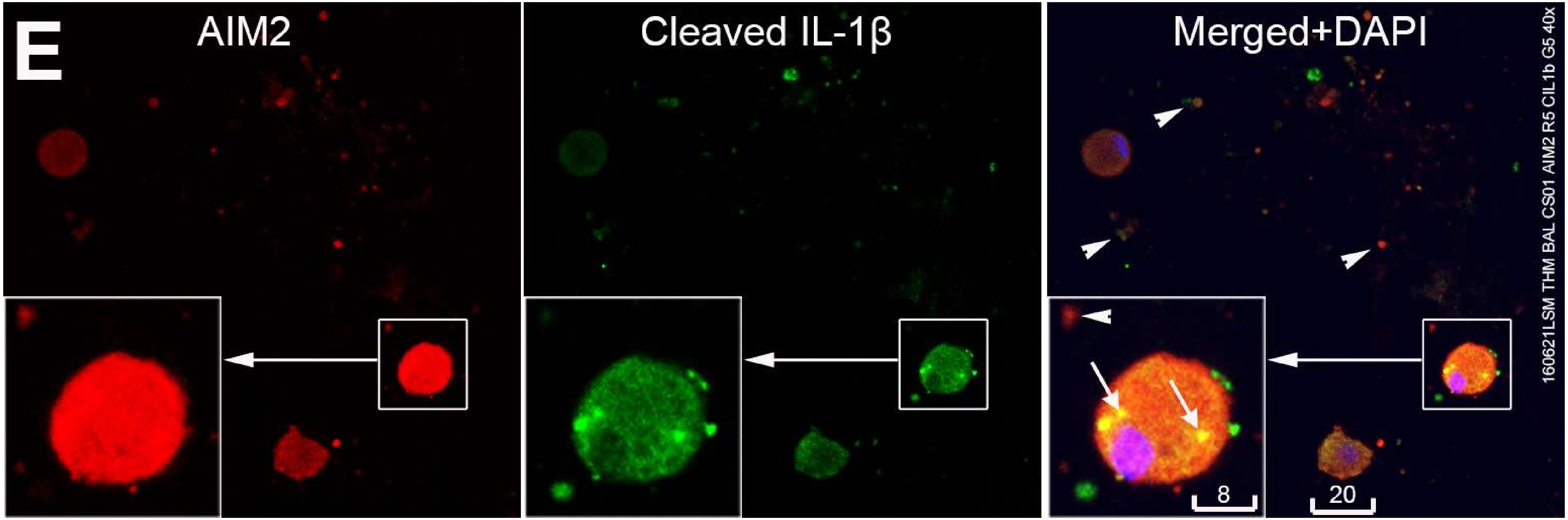

Figure S7. Representative confocal image of cigarette smoke extract-treated alveolar macrophages, showing intracellular (short arrows) and extracellular (arrowheads) of cleaved IL-1β particles colocalized with AIM2. Blue is DAPI. Scale bars are in micrometers.
